# Forks on the Run: Can the Stalling of DNA Replication Promote Epigenetic Changes?

**DOI:** 10.3389/fgene.2017.00086

**Published:** 2017-06-22

**Authors:** Hollie Rowlands, Piriththiv Dhavarasa, Ashley Cheng, Krassimir Yankulov

**Affiliations:** Department of Molecular and Cellular Biology, University of Guelph, GuelphON, Canada

**Keywords:** DNA replication, nucleosome assembly, histone chaperones, replication fork, replication pausing, replication fork barriers

## Abstract

Built of DNA polymerases and multiple associated factors, the replication fork steadily progresses along the DNA template and faithfully replicates DNA. This model can be found in practically every textbook of genetics, with the more complex situation of chromatinized DNA in eukaryotes often viewed as a variation. However, the replication-coupled disassembly/reassembly of chromatin adds significant complexity to the whole replication process. During the course of eukaryotic DNA replication the forks encounter various conditions and numerous impediments. These include nucleosomes with a variety of post-translational modifications, euchromatin and heterochromatin, differentially methylated DNA, tightly bound proteins, active gene promoters and DNA loops. At such positions the forks slow down or even stall. Dedicated factors stabilize the fork and prevent its rotation or collapse, while other factors resolve the replication block and facilitate the resumption of elongation. The fate of histones during replication stalling and resumption is not well understood. In this review we briefly describe recent advances in our understanding of histone turnover during DNA replication and focus on the possible mechanisms of nucleosome disassembly/reassembly at paused replication forks. We propose that replication pausing provides opportunities for an epigenetic change of the associated locus.

## Introduction

In eukaryotes, the advancement of replication forks is coupled to the disassembly of chromatin and its reassembly on the new DNA helices. For the most part, the pre-existing epigenetic marks are transmitted to the reassembled chromatin to confer its preservation and propagation. At the same time, changes in the epigenetic state of numerous loci are key events during cell differentiation and the development of metazoan organisms, during carcinogenesis, during plant and pathogen adaptation (Young, 2011; Alabert and Groth, 2012; Wyse et al., 2013; Yankulov, 2013; Almouzni et al., 2014). The mechanisms of such epigenetic changes are not well understood.

More than 1400 transient replication pause sites have been reported in the small genome of *Saccharomyces cerevisiae* (Ivessa et al., 2003; Makovets et al., 2004; Azvolinsky et al., 2009). These sites include subtelomeric DNA, *tRNA* genes, *rRNA* genes, highly transcribed protein-encoding genes, dormant origins of DNA replication, gene silencers, centromeres and secondary DNA structures, such as G4 quadruplexes. Given that 200–400 origins fire during S-phase (Raghuraman et al., 2001; Nieduszynski et al., 2006; Hawkins et al., 2013), each replication fork would normally encounter one to three such pausing sites. Similar frequency of replication pausing is expected in the cells of multicellular organisms. For example, 360,000 putative G4-forming elements have been identified in the human genome (Huppert and Balasubramanian, 2005) and multiple sites of fork stalling can be observed upon deprivation of dNTPs or histones (Lambert and Carr, 2013; Khurana and Oberdoerffer, 2015). However, the complexity of metazoan genomes and the significant heterogeneity of replicon sizes in different cell types (Berezney et al., 2000; Almouzni and Cedar, 2016) suggest that the stalling of forks in metazoan cells could vary both between cells and between different regions of the genome.

The stalling of replication forks opens up the risk of fork collapse and damage to DNA. To prevent such adverse effects, cells engage a variety of factors that stabilize the paused forks and aid the timely resumption of elongation. It is well established that mutations in such factors or artificially prolonged fork arrest can lead to checkpoint activation and genome instability. This topic has been extensively studied and reviewed (Putnam et al., 2012; Khurana and Oberdoerffer, 2015; Polo and Almouzni, 2015; Ang et al., 2016) and is not discussed here.

It is also possible that replication stalling could affect the replication-coupled turnover of chromatin and, consequently, could predispose adjacent loci to epigenetic changes. However, limited information on the fate of histones at paused replication forks is available. In this manuscript, we briefly review the current knowledge on the transmission of epigenetic marks during DNA replication and discuss the possibility of perturbations to nucleosome disassembly/reassembly at transient replication pausing sites. We suggest that the pausing of replication forks provides a window of opportunity for a change in the epigenetic state of a locus.

## Duplication of DNA and Chromatin

### DNA Replication

During DNA replication, DNA polymerases carry out DNA synthesis in a semi-conservative manner to produce two copies of the existing double helix. Many additional factors work concurrently with the polymerases to ensure the high fidelity and processivity of DNA replication. To guarantee that only one round of DNA replication occurs in each cell cycle, pre-replicative complexes are formed in G1 phase to “license” certain genome positions as origins. In S-phase, CDKs activate these “licensed” complexes to fire only once. Upon licensing the MCM complex is converted to the active CMG (Cdc45-MCM-GINS) helicase to unwind DNA and to form replication forks (Masai et al., 2010). Elongation factors are then recruited, many of them via interactions with the core homo-trimeric sliding clamp PCNA (Mailand et al., 2013). The CMG helicase moves ahead of the forks and generates DNA supercoiling ahead of the fork as well as catenation of the newly synthesized DNA strands behind the fork. Topoisomerases cut double stranded DNA to relieve the supercoiling and to catalyze the decatenation of the DNA duplexes (Schalbetter et al., 2015).

### Replication Stress and Pausing of the Forks

The term “replication stress” refers to various impediments, which cause the slowing down or the pausing of the replication forks (Zeman and Cimprich, 2014; Khurana and Oberdoerffer, 2015; Nikolov and Taddei, 2016). Replication stress can be caused by DNA damage, by deprivation or imbalance of nucleotides or by insufficient supply of histones. In addition, replication stress can be induced by tightly bound non-histone proteins, the collision of replication and transcription complexes or secondary DNA structures (Lambert and Carr, 2013; Zeman and Cimprich, 2014; Khurana and Oberdoerffer, 2015). The latter impediments have been identified as transient replication pausing sites during the normal course of DNA replication (Ivessa et al., 2003; Makovets et al., 2004; Azvolinsky et al., 2009).

At transient pausing sites the forks are stabilized against topological stress and collapse by at least two factors, the FPC and the cohesin-like Smc5/6 complex (Menolfi et al., 2015; Bastia et al., 2016). In addition, components of the replisome and the histones in the vicinity of the stalled forks undergo specific PTM (Baker et al., 2010; Szilard et al., 2010; Bastia et al., 2016). DNA helicases are then recruited (or activated) to aid the removal of the impediments and help the resumption of elongation. In *S. cerevisiae*, the Rrm3p helicase is engaged in the displacement of tightly bound non-histone-proteins (Ivessa et al., 2003) while Pif1p and Srs2p are involved in the unwinding of G4 quadruplexes or DNA hairpins (Anand et al., 2011; Paeschke et al., 2013; Leon-Ortiz et al., 2014). These helicases are not essential as their destruction does not prevent the completion of S-phase; however, their loss leads to extended stalling and increased mutation rates (Putnam et al., 2012; Ang et al., 2016). Similar DNA helicases (FANCJ, WRN, BLM) are found in metazoans and again are linked to higher mutation rates and cancer (Sarkies and Sale, 2012a). Hence, the compromised pausing of replication forks generates mutations and genome instability.

Replication pausing also seems to interfere with the stable transmission of chromatin (Sarkies and Sale, 2012b; Khurana and Oberdoerffer, 2015), but our understanding of these processes is limited. For example, multiple protein-binding sites and G4 structures contribute to frequent fork pausing in the subtelomeric regions of *S. cerevisiae* that is exacerbated upon deletion of the *RRM3* gene (Ivessa et al., 2003; Makovets et al., 2004; Azvolinsky et al., 2006). Interestingly, subtelomeric genes also undergo spontaneous epigenetic conversions, a phenomenon referred to as Telomere Position Effect (Gottschling et al., 1990; Yankulov, 2013). Similarly, the *rRNA* gene array of *S. cerevisiae* contains *RFB* (Replication Fork Barrier) sites, which arrest forks and prevent their collisions with transcription complexes (Kaplan and Bastia, 2009). The *rRNA* gene repeats are also subjected to spontaneous epigenetic conversions (Kaplan and Bastia, 2009; Yankulov, 2013). In chicken cells, epigenetic instability has been linked to the pausing of replication at G4 quadruplex-forming structures and can be exacerbated by depletion of nucleotides and by the deletion of the FANCJ gene (Schwab et al., 2013, 2015; Schiavone et al., 2014; Papadopoulou et al., 2015). It appears from this information that the pausing of replication forks is linked to epigenetic conversions, but the underlying mechanisms are not well understood.

### Duplication and Preservation of Chromatin

Before we address the possible effect of transient replication pausing on the preservation of chromatin, we will briefly summarize the current knowledge on the transmission of epigenetic marks during the advancement of the forks. The transmission of the methylation marks on DNA has been reviewed by others (Alabert and Groth, 2012; Almouzni and Cedar, 2016) and will not be discussed here. The disassembly and reassembly of nucleosomes is mediated by a complex network of histone chaperones, nucleosome remodelers and histone modifying enzymes. Many of these factors work in close contact with the basal replication machinery (Alabert and Groth, 2012; Gurard-Levin et al., 2014; Almouzni and Cedar, 2016). The overall process of disassembly, histone chaperoning and reassembly seems highly conserved between eukaryotes. However, the histone modifying enzymes, PTM and the timing of their restoration differ between organisms (Alabert and Groth, 2012; Annunziato, 2015). In the following sections we focus on the interactions between histones and their chaperones and discuss how replication pausing can alter the reassembly of nucleosomes.

#### Disassembly of the Nucleosomes

**FIGURE 1:**
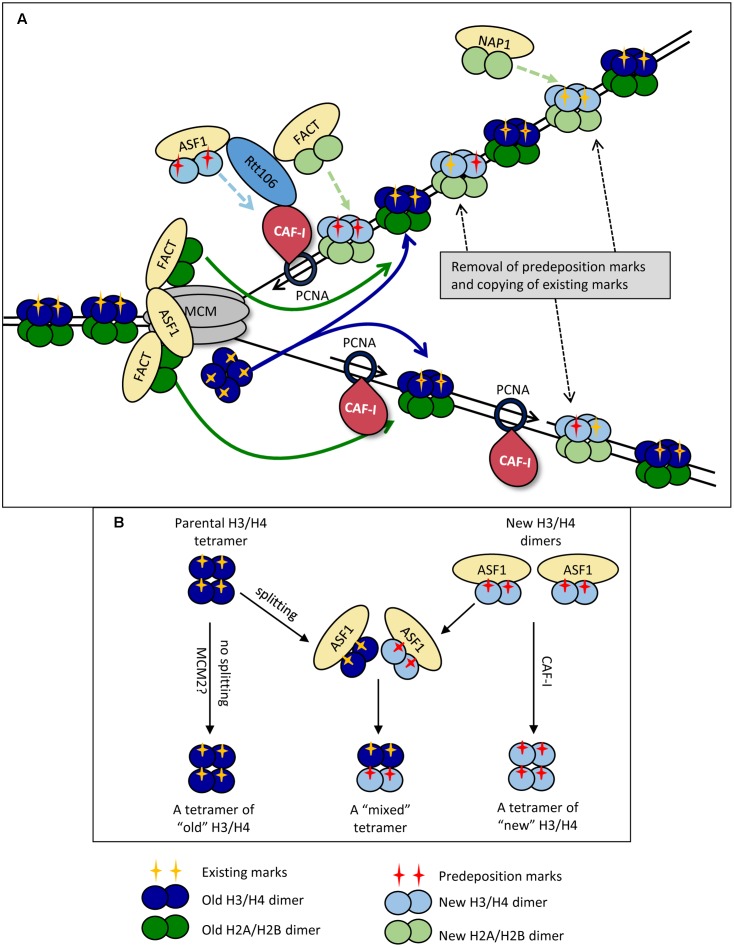
Replication-coupled disassembly and reassembly of nucleosomes. **(A)** Disassembly and reassembly during elongation. FACT disassembles H2A/H2B dimers (dark green) and transports them behind the fork, where it assembles them onto the new DNA strands. FACT also transports and assembles “new” H2A/H2B dimers (light green) onto the new DNA strands. Nap1p may play a role in the assembly of new H2A/H2B behind the fork in a manner not directly coupled with the movement of the replisome. ASF1 delivers newly synthesized dimers of H3/H4 (light blue) bearing pre-deposition PTMs (red cross) to the fork. “Old” H3/H4 histones (dark blue) bearing the existing PTMs (yellow cross) are disassembled and transported behind the fork as tetramers. The ferrying of old and new histones is indicated by colored solid and broken line arrows, respectively. CAF-I associates with PCNA and assembles H3/H4 tetramers, but may also be involved in the reassembly of “old” H3/H4. “Old” and “new” H3/H4 tetramers are randomly deposited on the leading and lagging strands. Rtt106p is involved in the delivery of new H3/H4 histones, but also interacts with ASF1, CAF-I and FACT and may coordinate the assembly of H3/H4 and H2A/H2B. At varying time points after the passage of the fork, the existing he PTMs of “old” H3/H4 (yellow cross) are read by histone modifying complexes and copied onto the adjacent new H3/H4 while pre-deposition marks (red cross) are erased. **(B)** Dimer versus tetramer H3/H4 transfer models and the transmission of histone marks. Old H3/H4 tetramers are transferred (possibly by MCM2) and deposited onto the newly synthesized strand without splitting (Left). Newly synthesized H3/H4 dimers are delivered by ASF1 and assembled by CAF-I (Right). At certain positions of the genome or upon replication stress the old H3/H4 tetramers are split by ASF1, mixed with new H3/H4 dimers and re-assembled into mixed tetramers (center). For details see Almouzni and Cedar (2016).

It is not known precisely how the replication-coupled histone chaperones are recruited and loaded on the fork. However, their subsequent activity is reasonably well understood (Alabert and Groth, 2012; Almouzni and Cedar, 2016). Current models suggest the disassembly of nucleosomes is executed by the chaperones FACT and ASF1, which tether to the CMG helicase complex in front of the fork (Groth et al., 2007; Abe et al., 2011; Wang et al., 2015) (**Figure 1A**).

H2A and H2B dimers are removed first by FACT (Abe et al., 2011; Winkler et al., 2011; Hondele et al., 2013; Yang J. et al., 2016) (**Figure 1A**). It is well established that H2A/H2B histones interact with FACT as dimers, and are most stable in this form (Abe et al., 2011). The position of the H2A/H2B contact with the Spt16p of FACT overlaps with the H2A/H2B contact with DNA and is likely to contribute to the release of H2A/H2B from DNA (Winkler et al., 2011; Hondele et al., 2013). Importantly, up to two FACT complexes can simultaneously interact with one nucleosome suggesting that histones H2A and H2B are disassembled and ferried behind the fork as dimers (Winkler et al., 2011). More recent studies have identified that FACT also has an affinity for H3 and H4, though it is unclear whether its affinity is greater for H2A/H2B or H3/H4 (Winkler et al., 2011; Hondele et al., 2013; Yang J. et al., 2016). It is plausible that the interactions with H3/H4 allow for further conformational changes within the octamer and aid the disassembly of the nucleosome as a whole (Winkler et al., 2011; Yang J. et al., 2016). In this line of thinking, it is conceivable that FACT contributes to the trafficking of H3/H4 behind the fork, but such a role is yet to be characterized.

Anti-silencing factor 1 also associates with CMG via the Mcm2p subunit (Groth et al., 2007; Wang et al., 2015). It removes H3/H4 tetramers after the release of H2A/H2B (Daganzo et al., 2003; Adkins et al., 2007; Natsume et al., 2007) (**Figure 1A**). It is well established that H3/H4 contact ASF1 with the globular domain of H3, with secondary contacts on the H4 tail (Daganzo et al., 2003; English et al., 2006; Natsume et al., 2007). Of particular importance, it has been determined that the site of association of ASF1 with H3 is at a position, which directly overlaps the interface at which the (H3/H4)_2_ tetramer is formed (Luger et al., 1997; English et al., 2005, 2006). This precludes the possibility that ASF1 associates with H3/H4 tetramers. Additional support for this idea comes from static light scatter assays with ASF1 and H3/H4, which showed that ASF1 interacts with H3/H4 dimers and not tetramers (Natsume et al., 2007). These observations suggest that the H3/H4 tetramer is split during the disassembly process. However, there is a solid evidence that the majority of H3/H4 tetramers do not split during DNA replication (Xu et al., 2010; Katan-Khaykovich and Struhl, 2011) thus questioning the precise role of ASF1. This issue has been revisited in a recent study, which suggests that the MCM complex itself (via its MCM2 subunit) can act as a chaperone and can hijack the H3/H4 tetramer interaction sites used by the nucleosomal DNA (Huang et al., 2015). In agreement with earlier observations (Xu et al., 2010; Katan-Khaykovich and Struhl, 2011), this activity of MCM2 can facilitate the ferrying and deposition of un-split H3/H4 tetramers (Clement and Almouzni, 2015). Even more, a direct transfer of the H3/H4 tetramer by MCM could ultimately occur without the participation of ASF1. For this reason it has been suggested that ASF1 could be necessary at positions of transient replication-fork barriers (including telomeres) or during replication stress (Clement and Almouzni, 2015) and not necessarily during unperturbed elongation. A simplified representation of the possible splitting of old H3/H4 tetramers by ASF1 at specific genome locations and the subsequent formation of “mixed” H3/H4 tetramers is shown in **Figure 1B**. ASF1 is also required for the trafficking of newly synthesized H3/H4 dimers to the nucleus (Blackwell et al., 2007) (**Figure 1A**).

Current models imply that after charging themselves with the disassembled histones, FACT and ASF1 dissociate from CMG and move behind the fork (Alabert and Groth, 2012; Almouzni and Cedar, 2016). This situation necessitates a constant pool of free chaperones in proximity to CMG that would replace the departed FACT and ASF1 and participate in the disassembly of the next nucleosome (**Figure 1A**). As mentioned above, it is not clear if the ASF1/FACT mode of transmission operates throughout the genome or only at specific positions.

#### Reassembly of the Nucleosomes

H3/H4 histones are the first histones deposited onto the new DNA strands and make a large contribution to the structural integrity of the nucleosome (Ray-Gallet et al., 2011; Gurard-Levin et al., 2014; Hainer and Martens, 2016) (**Figure 1A**). At present, CAF-I is the only factor that has been directly shown to assemble H3/H4 histones during DNA replication *in vitro* (Verreault et al., 1996; Shibahara and Stillman, 1999; Rocha and Verreault, 2008). In yeast, it is composed of three subunits Cac1p, Cac2p and Cac3p, and this structure is highly conserved across eukaryotes (Rolef Ben-Shahar et al., 2009; Jeffery et al., 2015). CAF-I associates with the fork via its interaction with the sliding clamp PCNA and separately with DNA through a winged helix domain in its Cac1p subunit (Shibahara and Stillman, 1999; Zhang et al., 2016) (**Figure 1A**). CAF-I interacts with H3/H4 in a way that overlaps with the site of their association with DNA, which is thought to prevent aberrant H3/H4 association with DNA (Kim et al., 2016). In addition, CAF-I interacts with other chaperones engaged in the delivery/assembly of new histones. Specifically, it is known that in *S. cerevisiae* ASF1 enhances the acetylation of H3K56, which in turn enhances the binding of H3/H4 to Rtt106 and CAF-I (Recht et al., 2006; Tsubota et al., 2007). Rtt106p also physically interacts with the Cac1p subunit of CAF-I and with FACT (Huang et al., 2005; Li et al., 2008; Fazly et al., 2012; Yang J. et al., 2016). It has been hypothesized that Rtt106p collaborates with CAF-1 and FACT and coordinates the replication-coupled nucleosome assembly of H3/H4 and H2A/H2B (Yang J. et al., 2016) (**Figure 1A**). Of note, an Rtt106 homolog has not been identified in mammals so the coordination of nucleosome assembly in these organisms must be mediated by other chaperones.

There is some uncertainty on the precise role(s) of CAF-I. Many models assume that CAF-I deposits both old and new histones (Almouzni and Cedar, 2016), however, it has not been formally shown that CAF-I interacts with “old” H3/H4. An alternative view suggests that CAF-I is responsible for the deposition of new histones only (Sarkies and Sale, 2012b). In addition, our current understanding of the interactions of CAF-I, ASF1 and Rtt106p has led to the idea that H3/H4 histones are deposited by CAF-I as tetramers, but direct evidence is yet to be obtained (Alabert and Groth, 2012). What is less well understood is the final composition of these tetramers (Almouzni and Cedar, 2016). For example, if CAF-I is indeed involved in the reassembly of old histones and the H3/H4 tetramers are split into dimers upon ASF1-driven disassembly, the splitting of the tetramer could be only transient, with Rtt106 facilitating re-tetramerization prior to assembly by CAF-I (Daganzo et al., 2003; Fazly et al., 2012; Kim et al., 2016). Following the deposition of H3/H4, the H2A/H2B dimers are assembled into the nucleosome. As the only identified H2A/H2B chaperone that directly interacts with fork components, FACT is assumed to be responsible for the replication-coupled reassembly of H2A/H2B (Gurard-Levin et al., 2014; Yang J. et al., 2016) (**Figure 1A**). At the same time, another histone chaperone, NAP1, could deposit H2A/H2B without a direct connection with the replication fork (Seebart et al., 2010; Almouzni and Cedar, 2016) (**Figure 1A**).

In summary, it seems that the re-assembly of the nucleosomes is centered at the CAF-I/PCNA “hub” behind the fork. It is distinct from the nucleosome disassembly “hub” formed by FACT, ASF1 and the CMG helicase ahead of the fork (**Figure 1A**). It is therefore plausible that the disruption of helicase-polymerase coordination could also affect the transmission of the existing epigenetic marks and reassembly of chromatin in the wake of the fork.

#### Timing of Reconstitution of Histone PTMs

The reassembly of nucleosomes in the wake of the fork involves the delivery of new histones and their incorporation along with the old histones. The new histones carry predeposition marks. On H3 and H4, these are acetylated lysines at varying positions in different species (Alabert and Groth, 2012; Bhaskara et al., 2013; Nagarajan et al., 2013; Khurana and Oberdoerffer, 2015). These PTMs facilitate the transfer of the H3/H4 between histone chaperones and their assembly onto the newly synthesized DNA, as described earlier for the H3K56 acetylation in *S. cerevisiae* (Recht et al., 2006; Tsubota et al., 2007). Upon deposition, some of the new histones can also be specifically modified. For example, in human cells H4K20 is mono-methylated very soon after replication and this modification is required for the subsequent deacetylation of this histone (Scharf et al., 2009b). The histone predeposition marks are eventually erased from chromatin, but the timing of removal can vary in different regions of the genome. An earlier study has demonstrated that the H4K5Ac/H4K12Ac predeposition marks are removed 20–60 min after the reassembly of heterochromatin while this delay was not seen in euchromatin regions (Taddei et al., 1999). Interestingly, recent studies have shown that suppression of removal of predeposition marks reduces the velocity of replication forks and can lead to replication stress (Bhaskara et al., 2010, 2013; Wells et al., 2013). It is tempting to speculate that the removal of some predeposition marks takes place soon after the passage of the fork and the slowing of the forks is produced by the failure to do so.

In general, most of the pre-existing histone marks are transmitted with the parental histones to the newly replicated DNA and are copied onto the new histones within one cell cycle (Scharf et al., 2009a; Alabert et al., 2015) (**Figure 1A**). However, specific PTMs display different kinetics of reconstitution. It has been reported that in human cells post-replicative histone acetylation and deacetylation is very dynamic, that a burst of mono-methylation of H3 and H4 takes place soon after replication while di- and tri-methylation shows a slower reconstitution (Scharf et al., 2009a). Even more, the maturation of some specific modifications (H3K9me3 and H3K27me3) can extend beyond one generation (Xu et al., 2012; Alabert et al., 2015). In this regard, it is worthwhile mentioning that some Histone-Methyl-Transferases of Histone-Acetyl-Transferases directly interact with fork components suggesting that they act during or immediately after the passage of the fork (Meijsing and Ehrenhofer-Murray, 2001; Sarraf and Stancheva, 2004; Huen et al., 2008; Reiter et al., 2015). On the other hand, recent analyses of chromatin turnover in *Drosophila* cells indicate that certain pre-existing marks, such as H3K4me3 and H3K27me3 are erased during S-phase. In parallel, the enzymes responsible for these PTMs are quickly re-loaded onto the new chromatin where they re-instate the marks after the completion of S-phase or even after cell division (Abmayr and Workman, 2012; Petruk et al., 2012, 2013). However, in *Caenorhabditis elegans* the methylation of H3K27 is retained following DNA replication of the repressed X chromosome (Gaydos et al., 2014). The emerging picture is that many, but not all, of the epigenetic marks on the “old” histones are maintained during the disassembly/reassembly process. Many of these marks serve as the carriers of epigenetic information for the rebuilding of chromatin and are eventually copied onto the new histones. However, the timing and the mechanisms of the restoration of specific marks show significant variations (**Figure 1A**).

## What Happens when the Fork Stalls?

The transient pausing of replication forks can be caused by multiple impediments and is not uncommon. Stalled forks are stabilized against topological stress and collapse and specific factors are recruited or activated to resume elongation. In the following sections we will discuss each of these aspects of fork pausing and their possible impact on the preservation of epigenetic marks.

### Fork Distortion and the Prevention of Topological Stress

**FIGURE 2:**
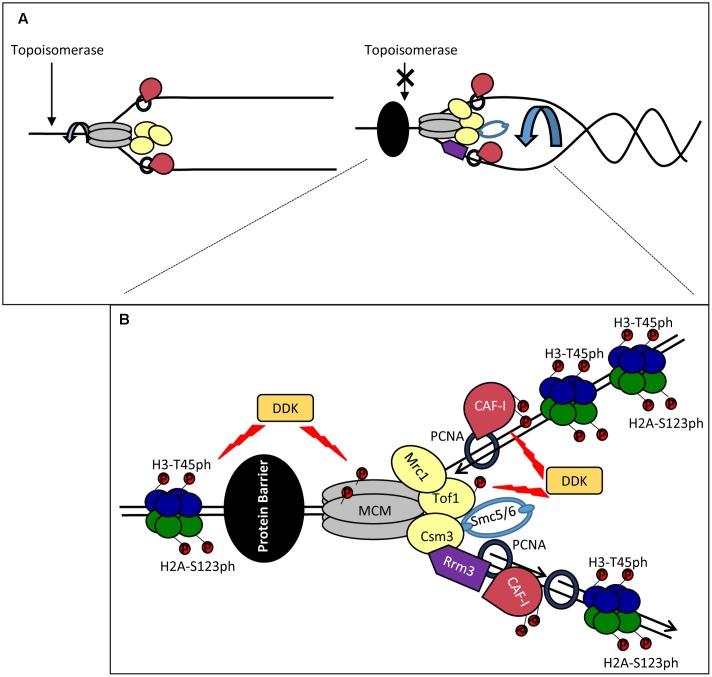
Stalling of a replication forks: distortion, stabilization and restarting. **(A)** Fork catenation and distortion at paused replication forks. During elongation, supercoiling and fork rotation are relieved by topoisomerases and the FPC (Left). The action of topoisomerases are inhibited by impediments (a non-histone DNA-binding protein is shown by a black oval), resulting in fork rotation and catenation (Right). **(B)** Stabilization and phosphorylation of fork components. The paused replisome is stabilized by the FPC (Mrc1p, Tof1p and Csm3p) which associates via the CMG helicase. DDK phosphorylates (red circles) MCM and Tof1p and contributes to fork stability. Other potential DDK targets include CAF-I and H3T45. H2A-S123 phosphorylation also occurs at a paused replication fork but the kinase is unknown. Rrm3p (purple) associates with PCNA on the lagging strand and removes the tightly bound protein via 5′-3′ helicase activity. Smc5/6 (light blue) cooperates with Rrm3p and FPC and counteracts prolonged pausing caused by Tof1p-Csm3p. For simplicity, new histones, Asf1, FACT and Rtt106 are not shown. It is not established if the histones are phosphorylated ahead of or behind the fork.

The advancing CMG helicase generates extensive negative supercoiling of the replicated DNA ahead of the fork. In turn, this torsional stress can force fork rotation and double-stranded catenanes behind the fork (**Figure 2A**). During elongation, the DNA supercoiling is relieved by topoisomerases, while fork rotation is suppressed at least in part by the so-called FPC (**Figure 2A**). FPC is composed of three proteins, Mrc1p, Tof1p and Csm3p (Bando et al., 2009; Komata et al., 2009) and is believed to be part of the elongating replisome (Gambus et al., 2006; Komata et al., 2009). *TOF1* and *CSM3* are required for the stabilization of replication forks in the presence of hydroxyurea (Bando et al., 2009) and at sites of tightly bound non-histone proteins (Mohanty et al., 2006; Tourriere and Pasero, 2007). Mrc1p is required for the normal progression rate of DNA replication forks and for the activation of checkpoints, but is dispensable for pausing (Tourriere et al., 2005; Mohanty et al., 2006; Hodgson et al., 2007; Petermann et al., 2008). Tof1p-Csm3p directly associate with the CMG helicase via Mcm2p while the association of Mrc1p is dependant on Tof1p-Csm3p (Bando et al., 2009) (**Figure 2B**).

A recent study has demonstrated increased catenation of the fork at several types of replication pausing sites (Schalbetter et al., 2015). It has been suggested that these effects are caused by the interference of the impediment (a tightly bound protein or a secondary DNA structure) with the activity of the topoisomerases. At these positions, Tof1p-Csm3p prevents further topological stress until the impediment (in this situation a non-histone protein) is removed by the displacement helicase Rrm3p (**Figure 2B**). In support of this idea, replication pausing is diminished upon the deletion of *TOF1* or *CSM3* (Hodgson et al., 2007) and fork rotation is exacerbated in the absence of *TOF1*, *CSM3* and *RRM3* (Schalbetter et al., 2015). Other factors are also involved in the coordinated activity of Rrm3p and Tof1p-Csm3p. It has been recently found that the cohesin-like Smc5/6 complex is enriched at sites of replication pausing and overlaps with sites of Rrm3p enrichment (Menolfi et al., 2015; Branzei and Menolfi, 2016). Notably, it was shown that Smc5/6 cooperates with Rrm3p and counteracts the prolonged pausing caused by Tof1p-Csm3p (Menolfi et al., 2015). Hence, the torsional fork rotation can lead to a quick resolution of the arrest, but also to complications due to fork distortion and catenations.

In summary, replication stalling is accompanied by topological distortion of the forks, which could lead to temporal perturbation of histone transmission and assembly. We discuss this possibility in Section “What Happens When the Fork Stalls?”.

### DDK (Dbf4-Dependent Kinase) and Its Possible Role at Paused Forks

Dbf4-Dependent Kinase is an essential kinase, which phosphorylates several MCM proteins (**Figure 2B**). Some of these phosphorylation events are critical for the firing of the origins (Sheu and Stillman, 2010). However, it has long been speculated that the function of DDK extends beyond the control of origins (Duncker and Brown, 2003). In budding yeast, DDK phosphorylates Tof1p-Csm3p and subunits of the MCM helicase at the stably arrested replication forks in the *rRNA* gene arrays (Kaplan and Bastia, 2009; Bastia et al., 2016) (**Figure 2B**). In turn, the phosphorylated Tof1p-Csm3p associates with MCM to inhibit its helicase activity and to counteract the activity of Rrm3p (Cho et al., 2013; Bastia et al., 2016) (**Figure 2B**). It is not known if the DDK-dependent phosphorylation of MCM is identical at origins and at the pausing site or if the phosphorylation of MCM precludes its possible activity as a histone chaperone (Clement and Almouzni, 2015; Huang et al., 2015). In human cells the homolog of Mrc1p, Claspin, directly associates with DDK (Yang C.C. et al., 2016). At this point it is uncertain if Claspin, which is recruited to stalled forks (Masai et al., 2010), could also engage DDK during fork pausing.

Dbf4-Dependent Kinase also phosphorylates CAF-I in both budding yeast and human cell extracts (Keller and Krude, 2000; Gerard et al., 2006; Jeffery et al., 2015) (**Figure 2B**). In human cell extracts the p150 subunit of CAF-I can form dimers (Gerard et al., 2006). The phosphorylation of p150 by DDK prevents this dimerization and stimulates its binding to PCNA (Gerard et al., 2006). However, both monomeric and dimeric forms of p150 seem to be a requirement for CAF-I activity. On the other hand, in *S. cerevisiae* the phosphorylation of p150 is not necessary for the loading of CAF-I to chromatin, suggesting that these events can take place at a later stage of DNA replication (Jeffery et al., 2015).

Finally, DDK phosphorylates the Histone H3T45 residue (Baker et al., 2010). This phosphorylation peaks in S-phase and H3-T45A mutations reduce the resistance of cells to hydroxyurea and camptothecin (an inhibitor of Topoisomerase I), but not to DNA alkylating agents (Baker et al., 2010). Importantly, H3T45 phosphorylation is not required for the initiation of DNA replication, but is necessary at a later step (Baker et al., 2010). Histone H2A is also phosphorylated at *RRM3*-dependent transient replication pause sites, but the actual kinase has not been identified (Szilard et al., 2010) (**Figure 2B**). Interestingly, in human cells gamma-H2AX accumulates at hydroxyurea-induced pause sites long before fork collapse and DNA damage, suggesting that this modification could be a regular event at transiently paused forks and not restricted to the DNA damage response (Sirbu et al., 2011).

It is conceivable that DDK is recruited to paused forks where it phosphorylates H3T45, Tof1p, Cac1p and the MCM complex (**Figure 2B**). The phosphorylation of Tof1p and MCM could prevent the premature resolution of pausing (Bastia et al., 2016) and the trafficking of old H3/H4 tetramers (Clement and Almouzni, 2015; Huang et al., 2015). DDK can also potentially alter the activity of CAF-I (Gerard et al., 2006; Jeffery et al., 2015). H3-T45 is positioned at the site where DNA enters and leaves the nucleosome (Baker et al., 2010). Its phosphorylation will almost certainly reduce the nucleosome-DNA contact and could facilitate the resumption of elongation, but could also affect the disassembly of the nucleosomes. All these events can promote a substantially different mode of H3/H4 handling and reassembly at the stalled fork.

## Histone Turnover at Paused Replication Forks

As mentioned earlier, at a paused fork DNA experiences topological distortion and several fork-associated factors are phosphorylated. No matter the reason for the pause, we expect a histone-free region of DNA that is occupied by the impediment (**Figure 2B**). In addition, the pausing of the fork is unlikely an abrupt event. It has been suggested that the impediments inhibit topoisomerase action ahead of the fork (Schalbetter et al., 2015). If this is the case, we could expect a gradual build-up of supercoiling and retardation of the fork progression before it actually stalls. Under these conditions the disassembly/reassembly of more than one nucleosome can be affected. These few (or even one) odd nucleosomes can destabilize an existing array of similarly modified nucleosomes and provide the means for other factors to convert the epigenetic state of the locus. These possibilities are addressed below.

### The Fate of Old H3/H4 Histones

The first key consideration is the fate of the old H3/H4 histones at paused forks. We know that the majority of old H3/H4 tetramers are not split during DNA replication (Xu et al., 2010; Katan-Khaykovich and Struhl, 2011) and that MCM alone can act as a chaperone for the H3/H4 tetramer (Huang et al., 2015). However, ASF1 can only interact with H3/H4 dimers and H3/H4 dimers in complex with CAF-I have been identified in nuclear extracts (Tagami et al., 2004; Natsume et al., 2007). Importantly, during replication stress ASF1 is found in complex with H3/H4 with typical parental PTMs (Groth et al., 2007; Jasencakova et al., 2010). Therefore, we have to consider that ASF1 may have different modes of action during elongation and at paused forks. Consequently, two models are possible. Both of them envisage some loss of epigenetic marks.

**FIGURE 3:**
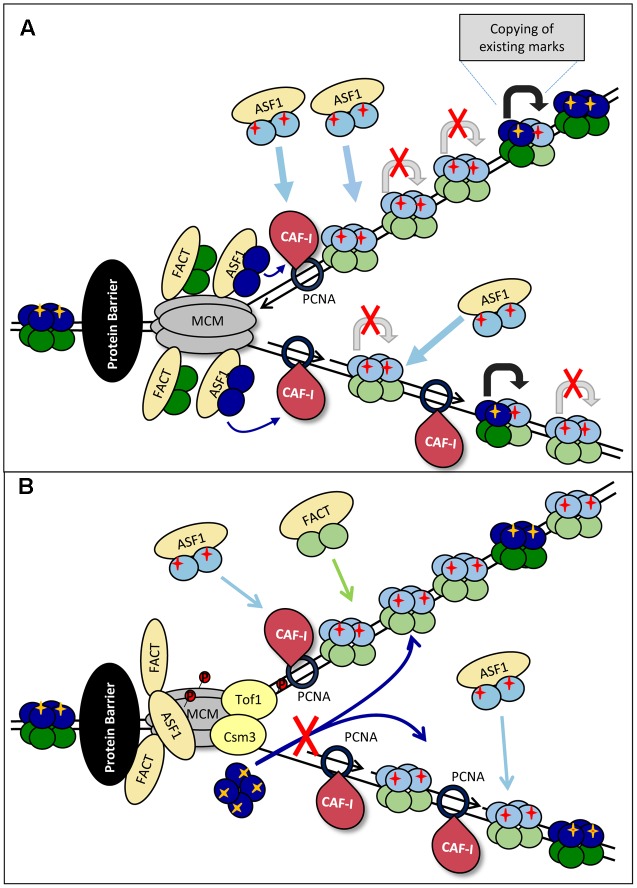
Models for loss of histone marks. **(A)** Splitting of the H3/H4 tetramer. Upon approaching the impediment (a non-histone DNA-binding protein is shown by a black oval) the forks are slowing down. “Old” H3/H4 tetramers (dark blue) are split into dimers by ASF1 and transported behind the fork and delivered to CAF-I to form mixed tetramers with the new H3/H4 histones (light blue). The PTMs on the “old” dimers (yellow cross) serve as templates for the transmission of the marks to the “new” dimers (shown by gray arrows) from which pre-deposition marks are erased. The slowing and eventual pausing of fork reduces the availability of “old” histones, resulting in a disproportionately high assembly of “new” histones and loss of histone PTMs. **(B)** Inhibition of tetramer transmission. The impediment (a non-histone DNA-binding protein is shown by a black oval) and fork pausing is accompanied by the phosphorylation of MCM by DDK. This phosphorylation inhibits MCM helicase activity, but also its putative H3/H4 chaperone activity. Consequently, the delivery of “old” tetramers is precluded and CAF-I assembles nucleosomes from newly synthesized H3/H4 only.

The simplest model suggests that during elongation whole tetramers are ferried behind the fork, but upon slowing-down and subsequent pausing the “old” tetramers are split into dimers by ASF1 (**Figure 3A**). This notion comes from the observed increase in the abundance of ASF1 in complex with “old” H3/H4 during replication stress (Jasencakova et al., 2010). The old H3/H4 dimers could be transferred behind the fork, complemented by new H3/H4 dimers and deposited on the new DNA strands as a mixed tetramer. In this case, the marks from the old histones could by copied on the new ones within the same nucleosome (**Figure 3A**). This model is in agreement with the previously proposed assembly of “mixed” nucleosomes in (Clement and Almouzni, 2015; Almouzni and Cedar, 2016) (**Figure 1B**). Under these conditions, the mere slowing-down of the fork could decrease the supply of old histones and enhance the assembly of nucleosomes from new histones. This situation can be exacerbated upon complete pausing.

Another possibility for loss of old H3/H4 could be the differential regulation of the MCM helicase during elongation and upon stalling (**Figure 3B**). In Section “DNA Replication” and **Figure 2B** we described the DDK-dependent phosphorylation of the MCM helicase and Tof1p-Csm3p at arrested replication forks. Recent studies also suggest that MCM2 can act as a chaperone for the H3/H4 tetramer (Clement and Almouzni, 2015; Huang et al., 2015). The phosphorylated Tof1p-Csm3p binds to MCM and is known to inhibit its helicase activity (Cho et al., 2013; Bastia et al., 2016), but it is unclear if the putative MCM H3/H4 chaperone activity is affected. It is open to conjecture that if MCM is responsible for the ASF1/CAFI-independent transmission of H3/H4 tetramers behind the fork (Clement and Almouzni, 2015; Huang et al., 2015), the loss of this activity will promote the assembly of nucleosomes from new histones only (**Figure 3B**).

**FIGURE 4:**
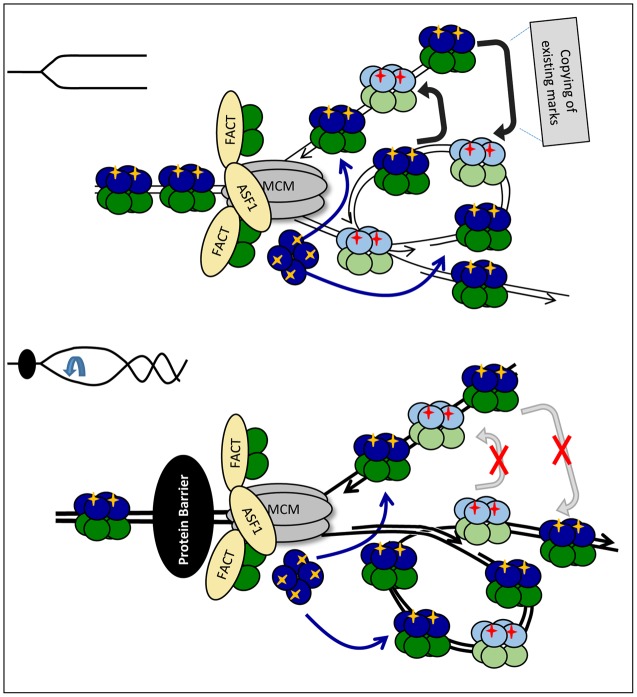
Fork rotation and loss of communication between leading and lagging strands. Upon approaching the impediment (a non-histone DNA-binding protein is shown by a black oval) H3/H4 histones are transferred and deposited onto the new strands as tetramers, but the fork is distorted by rotation and catenation (bottom). If some PTMs on the new histones are copied from the corresponding nucleosome on the sister DNA strand, the distortion of the fork will prevent the transmission of these marks. This loss of marks will come on top of the loss of marks due to the reduced availability of “old” H3/H4 as depicted in **Figure 3A**.

In the second model, ASF1 has a similar action during elongation and pausing. It transiently destabilizes old H3/H4 tetramers, but they quickly re-form before being deposited (randomly or not) on one of the new strands (**Figure 4**). The increased abundance of ASF1 complexed with old H3/H4 during replication stress represents this transitional state of the old H3/H4 histones. Under this scenario, the slowing-down of the fork and/or the inhibition of “old” H3/H4 transmission would still promote the deposition of new H3/H4, but an additional complication would exist: the new H3/H4 tetramers need to copy the existing marks from another nucleosome with an old H3/H4 tetramer. This nucleosome could be the neighboring one on the same strand or the corresponding one on the other strand (**Figure 4**). Intuitively, one would expect that the latter mechanism is sensitive to topological distortion and could be less conservative in the preservation of epigenetic state. As already described, transient fork pausing is associated with rotation that induces topological alterations (Schalbetter et al., 2015). These alterations could temporarily suspend the communication between the two nucleosomes, leading to loss of the pre-existing histone marks at that position (**Figure 4**).

Both models assume that the pausing of the fork affects the transmission of existing histone marks and early “seeding” PTMs that take place in the immediate wake of the fork. These seeding marks could subsequently influence the maturation of chromatin and the establishment of the marks with delayed kinetics of reconstitution (Scharf et al., 2009a; Alabert et al., 2015).

### The Role of CAF-I

It has been shown that both ASF1 and CAF-I remain associated with chromatin under conditions of transient arrest of DNA replication by hydroxyurea (Jasencakova et al., 2010; Petermann et al., 2010; Sirbu et al., 2011). While it is not given that this situation is identical at all kinds of pausing sites, it is likely that the key histone chaperones remain associated with the replisome at stalled forks. What would be the role of CAF-I at paused forks?

As discussed, it is assumed that CAF-I is responsible for the replication-coupled assembly of both old and new H3/H4 histones, but it is also possible that it works with new H3/H4 only (Sarkies and Sale, 2012b; Almouzni and Cedar, 2016). Consequently, two closely related mechanisms can describe the role of CAF-I at paused replication forks. Both mechanisms are based on the assumption that the increased abundance of ASF1 in complexes with old H3/H4, which are observed during replication stress (Jasencakova et al., 2010), reflects the decreased supply of old histones behind the fork. If CAF-I intercepts the old H3/H4 dimers from ASF1 (**Figures 3A,B**) and mixes them with new histones, the limiting supply of old histones would promote the assembly of nucleosomes with new histones only. If CAF-I does not intercepts old histones, it can continue to assemble new H3/H4 tetramers while the transmission of old H3/H4 by a CAF-I independent mechanism is temporarily suspended (**Figures 3A,B**). Both scenarios predict that in the absence of CAF-I the deposition of new histones, and concomitantly the probability for an epigenetic change, will be decreased. This conjecture is supported by the demonstrated reduction of epigenetic conversions upon the destruction of CAF-I in *S. cerevisiae* (Jeffery et al., 2013; Wyse et al., 2016).

It is also possible that the increased abundance of ASF1 complexes with old H3/H4 does not reflect a diminished delivery of old histones. In this situation the observed effects of CAF-I on epigenetic conversions could be explained by its altered activity upon stalling of the fork (**Figure 2B**). For example, we know that DDK phosphorylates CAF-I. In budding yeast this phosphorylation is not necessary for the association of CAF-I with chromatin, suggesting that DDK may act on CAF-I at a post-initiation event (Jeffery et al., 2015). Given the fact that DDK phosphorylates components of the stalled fork (Tof1p, MCM, H3T45, see above), it is not inconceivable that DDK specifically phosphorylates CAF-I at paused forks (**Figure 2B**). One possibility is that this phosphorylation event stimulates CAF-I activity toward new histones.

### Dissimilar Nucleosome Assembly on the Leading and Lagging Strands

**FIGURE 5:**
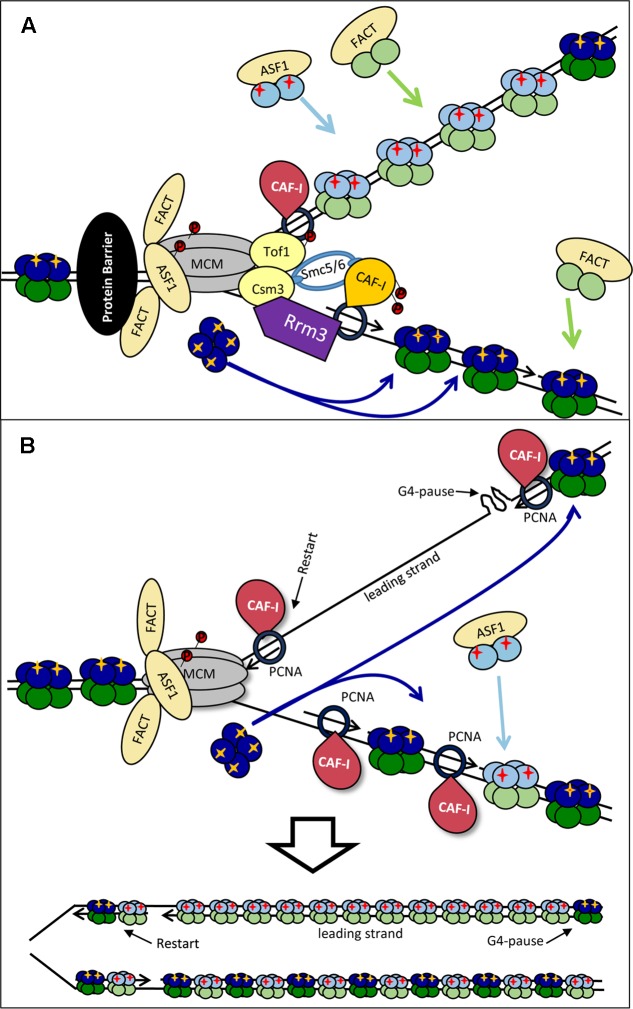
Models for the dissimilar nucleosome assembly on the leading and the lagging strands. **(A)** Modulation by Rrm3p. Rrm3p (purple) associates with PCNA on the lagging strand to displace a tightly bound protein (shown by a black oval). This Rrm3p–PCNA interaction alters the association of CAF-I (depicted by orange color) and precludes its activity on the lagging strand only. Consequently, the lagging strand intercepts “old” H3/H4 tetramers, but the assembly of new H3/H4 is inhibited. It is possible that the disproportional incorporation of “old” H3/H4 on the lagging strand promotes the deposition of newly synthesized histones on the leading strand. **(B)** By-passing of the impediment. A G4-DNA has arrested the DNA polymerase on the leading strand. DNA synthesis has restarted downstream of the impediment. The G4 structure is then relieved by a specialized helicase and the gap is filled in by a later stage DNA synthesis. However, this later-stage DNA synthesis takes place away from the source of “old” H3/H4 histones (dark blue) and nucleosomes are assembled with “new” histones (light blue) only. For details see Sarkies and Sale (2012b).

Another source of CAF-I modulation could be the PCNA-interacting proteins at stalled forks. Similarly to Cac1p, the budding yeast Rrm3p and Sgs1p helicases contain a PIP for the direct association with PCNA (Anand et al., 2011; Wyse et al., 2016). PCNA forms a homo-trimeric clamp capable of three PIP-mediated contacts, suggesting a complex communication between PCNA and its interacting partners (Mailand et al., 2013). It is not clear if Rrm3p is traveling with the fork or if it is recruited upon pausing (Calzada et al., 2005; Azvolinsky et al., 2006), however, it is assumed that it is activated on the lagging strand only (Ivessa et al., 2002) (**Figure 5A**). It is possible that the recruitment/activation of Rrm3p could alter the contact of PCNA with CAF-I or altogether displace CAF-I from PCNA (**Figure 5A**). This scenario puts forth the possibility that at paused forks Rrm3p imposes a different mode of reassembly on the lagging and leading strands and can grant the chance for a post-replicative epigenetic change on one of them only. In support of this idea, we have recently demonstrated that the deletion of *RRM3* suppressed epigenetic changes in *S. cerevisiae* (Wyse et al., 2016). The involvement of Rrm3p and the hypothesized suppression of CAF-I on the lagging strand suggests that the existing chromatin state can be altered on the leading strand where CAF-I continues to operate, but preserved on the lagging strand where its activity is reduced (**Figure 5A**).

Preferential loss of histone marks on one of the two strands can also be caused by the uncoupling of the synthesis of DNA on the leading and lagging strands as suggested in (Sarkies and Sale, 2012b) (**Figure 5B**). For example, a G4 quadruplex can form on the leading strand and arrest DNA synthesis on it while the CMG helicase continues to operate. Under these conditions the lagging strand will continue to initiate close to the fork and will therefore be exposed to the pool of disassembled “old” H3/H4 histones. On the leading strand DNA synthesis can restart downstream of the G4-block and the resulting single-strand gap could be filled-in at a later stage. However, this later stage of DNA synthesis would take place away from the source of old histones and CAF-I will reassemble nucleosomes with new histones only (Sarkies and Sale, 2012b; Papadopoulou et al., 2015) (**Figure 5B**, bottom).

In both cases, the dissimilar assembly of chromatin on one of the two strands would provide a mechanism where replication pausing would enhance the loss of old histones on the leading strand and thus generate the possibility of a genetic change on one of the chromatids only. Stem cells could use such mechanisms to maintain pluripotency and at the same time to generate differentiating progeny.

## Fork Pausing and Heterochromatinization

The described models imply that the transient pausing of replication forks leads to the erosion of epigenetic marks and generates the possibility for an epigenetic change. However, several studies have suggested that the fork stalling predominantly leads to heterochromatinization (Rivera et al., 2014; Nikolov and Taddei, 2016). For example, it has been shown in human cells that upon replication stress ASF1 is charged with mono-methylated H3K9 (Jasencakova et al., 2010), a mark that predisposes to tri-methylation, and the subsequent formation of heterochromatin (Loyola et al., 2006). In agreement, in *S. cerevisiae* the pausing of replication at an artificial site produced by *LacI* arrays induced the silencing of an adjacent reporter (Dubarry et al., 2011). Several other studies have demonstrated loss of gene activity that can be correlated to the prolonged pausing of replication forks (Nikolov and Taddei, 2016). However, other studies challenge the concept that newly assembled chromatin is silenced by default and that the pausing of replication exacerbates heterochromatinization.

One example is presented by the effect of expanded triplet repeats, which are known to form secondary DNA structure and impede replication. The insertion of such elements close to a reporter gene was shown to promote classical position effect variegation, regardless of the site of insertion (Saveliev et al., 2003). However, this effect has not been explicitly linked to the pausing of replication and other contributors to the variegation phenotype [such as the formation of R-loop structures during transcription (Schwab et al., 2013; Groh et al., 2014)] cannot be ruled out. Another study in chicken DT40 cells found that replication stalling at G4-quadruplex sites led to loss, rather than gain, of gene silencing and to the accumulation of new histones (Sarkies et al., 2010). In a follow-up study the same group showed that the insertion of G4-forming DNA near an active gene can lead to its deactivation (Sarkies et al., 2012). A study in *S. cerevisiae* also pointed out that the insertion of G4-DNA next to *URA3* or *CAN1* can produce variegation effects (Paeschke et al., 2013). It appears that a G4-forming DNA can drive epigenetic changes in both directions and can confer epigenetic instability rather than simply promoting heterochromatin formation.

Similarly, *RRM3*-dependent fork pausing at sites of tightly bound proteins has also demonstrated different effects on heterochromatinization and gene silencing. At multiple *LacI* arrays the silencing of the nearby reporter was enhanced by the deletion of *RRM3* (Dubarry et al., 2011). However, at telomeres the deletion of *RRM3* reduced the silencing of reporter genes (Ivessa et al., 2003). Our group has shown that the deletion of *RRM3* reduces the frequency of conversions of a sub-telomeric reporter from both silent-to-active and from active-to-silent states (Wyse et al., 2016).

The seemingly opposite effects in the listed reports could be reconciled if we assume that transient fork pausing at these positions can stimulate both silencing and anti-silencing. In other words, the pausing of the fork can expose the adjacent genes to epigenetic instability. We suggest that the observed locus-to-locus variation reflects complex mechanisms where the temporal disturbance of chromatin acts in synchrony with other activities that shape the state of the locus. For example, in the absence of dominating *cis*-elements the pausing of the fork would lead to the variegation phenotypes observed in (Ivessa et al., 2003; Saveliev et al., 2003; Jeffery et al., 2013; Paeschke et al., 2013; Wyse et al., 2016). However, at loci dominated by heterochromatin or euchromatin there would be no conversions regardless of the pausing event. In support of this idea, the mating type loci in *S. cerevisiae* are well-documented replication pausing sites, but never convert to active gene expression (Ivessa et al., 2002; Rusche et al., 2003; Makovets et al., 2004). This remarkable epigenetic stability can be attributed to the potent silencer *cis*-elements at these positions (Rusche et al., 2003). The same applies to the actively transcribed genes, which pause replication forks but are rarely, if at all, silenced (Azvolinsky et al., 2009). The situation can be different at sites of synthetic *LacI* arrays or at random sites of replication stress caused by the decline of dNTP pools or by deprivation of histones (Jasencakova and Groth, 2010; Jasencakova et al., 2010; Dubarry et al., 2011; Nikolov and Taddei, 2016). In such cases the deposition/exchange of mono-methylated H3K9 could serve a protective role.

In summary, it seems that G4-forming DNA, stem-loop DNA structures and tightly bound proteins all trigger epigenetic instability and variegation phenotypes rather than simply promoting gene repression. It remains to be established if the epigenetic instability is directly linked to their ability to pause replication forks.

## Replication Factories, Convenient Answers to Many Questions

The idea of replication factories is not new. The massive size of the replisome prompted the question of whether it moves along DNA or whether it is the DNA that is pulled through an immobilized replisome (Cook, 1999). In parallel, many studies have established that the estimated number of active origins by far exceeds the number of the observed replication foci (Berezney et al., 2000; Meister et al., 2006). For these reasons, it has been proposed that many replisomes cluster to form an immobile replication factory. The model of replication factories was further supported by the observation that two forks originating from a single origin do not separate during S-phase, suggesting that they remain associated with a “factory” (Meister et al., 2006). We can imagine that such factories are responsible for the duplication and reassembly of chromatin and that all the events of pausing happen within the factory.

Can some of the questions raised in this review be answered or extended by the existence of sophisticated “factories”? For example, the mating type loci, the telomeres and the *rRNA* gene clusters, which contain multiple pause sites, replicate late in the S-phase (Raghuraman et al., 2001). It is open to conjecture that “late” factories selectively work with late origins or that the factories are refitted in late S-phase. Such late factories need to accommodate Tof1p-Csm3p and Rrm3p, whose roles in normal elongation and pausing may not be the same, as well as the Pif1p and Sgs1p helicases. These factories may also recruit DDK and other kinases to dampen the deposition of new histones upon pausing of the forks. Finally, a steady supply of free ASF1 and FACT waiting in proximity to the fork is needed for the efficient resumption of chromatin disassembly. Many of these proteins interact with PCNA or with the MCM helicase (Alabert and Groth, 2012; Mailand et al., 2013; Almouzni and Cedar, 2016). We can imagine that there is a massive continuous rearrangement of the PCNA- and MCM-associating factors at each pause site or that all these events take place within a tightly controlled “factory.” For example, do the late forks operate in a factory where all of the mentioned factors reside in close proximity and can be immediately engaged/disengaged? In this situation, PCNA and MCM would be selecting/activating the acting factors without necessarily forming stable complexes with them. The real question is how the forks within this sophisticated “factory” are bent and modified to recognize and resolve the pausing. Another very important question is how chromatin is signaling to the factory to trigger these rearrangements.

## Concluding Remarks

The preservation of genetic information calls for the exceptionally high fidelity of DNA replication. Chromatin also provides transmissible information in the form of epigenetic marks. At the same time, chromatin commands a major regulatory role and, as such, its transmission should allow for alterations in gene expression and therefore for epigenetic change. In metazoans, epigenetic changes are the very foundation of cell differentiation and development (Almouzni and Cedar, 2016). In single-cell eukaryotes epigenetic changes help to adapt to changes in the environment (Wyse et al., 2013). After the establishment of a desired epigenetic landscape, the cells would preserve it by faithful transmission of the epigenetic marks.

The transmission and preservation of epigenetic marks has received significant attention. In comparison, the mechanisms of epigenetic changes are less studied and not so well understood. In this review we focused on the histone exchange at transient replication pausing sites. We propose that eukaryotic cells, in conjunction with other mechanisms, use such sites for controlled epigenetic conversions. If this is correct, research at the junction of epigenetics and DNA replication needs to be more intense.

## Author Contributions

HR, PD, and AC drafted the text and designed the figures. KY critically revised the manuscript and wrote and approved the final version.

## Conflict of Interest Statement

The authors declare that the research was conducted in the absence of any commercial or financial relationships that could be construed as a potential conflict of interest.

## Abbreviations

ASF1: Anti-Silencing Factor 1
CAF-I: Chromatin Assembly Factor 1
CDK: Cyclin Dependent Kinase
CMG: Cdc45-MCM- GINS
DDK: Dbf4-Dependent Kinase
FACT: Facilitator of Activated Transcription on Chromatinized Templates
FPC: Fork Protection Complex
MCM: Mini-Chromosome Maintenance
PCNA: Proliferating Cell Nuclear Antigen
PIP: PCNA-Interacting Peptide
PTM: Post-Translational Modifications.
